# Protocol of a prospective study for the combination treatment of Shu-Gan-jian-Pi decoction and steroid standard therapy in autoimmune hepatitis patients

**DOI:** 10.1186/s12906-016-1486-1

**Published:** 2016-12-07

**Authors:** Xiao-ling Chi, Huan-ming Xiao, Yu-bao Xie, Gao-shu Cai, Jun-min Jiang, Guang-jun Tian, Mei-jie Shi, Shu-duo Wu, Peng-tao Zhao, Hui-jun Chen

**Affiliations:** Department of Hepatology, Guangdong Provincial Hospital of Chinese Medicine, Guangzhou, 510120 China

**Keywords:** Autoimmune Hepatitis, Steroid, Side effects, Shu-Gan-Jian-Pi Decoction

## Abstract

**Background:**

Prednisone plus azathioprine is considered the mainstay of therapy in the current recommendations for autoimmune hepatitis (AIH). However, it does not provide good benefits for AIH patients because of its serious side effects. Therefore, more and more AIH patients prefer to seek for traditional Chinese medicine (TCM) to manage their symptoms and reduce the side effects of steroids in China. Shu-Gan-Jian-Pi Decoction is a popular used Chinese herbal formula in Guangdong province of China, which has demonstrated the effect of improving efficacy and reducing side effects of corticosteroids in AIH patients. The aim of this study is to evaluate the effects of Shu-Gan-Jian-Pi Decoction combined with steroid in AIH patients. So, this study aims to explore whether the combination treatment of Shu-Gan-Jian-Pi Decoction and steroid standard therapy could improve the clinical management of AIH.

**Methods:**

A prospective non-randomized study on AIH will be conducted between October 2015 and June 2017 in Guangdong Provincial hospital of Chinese medicine. Eligible AIH patients will be classified as the case group (*n* = 66) and the control group (*n* = 66) based on the interventions. Patients taking Shu-Gan-Jian-Pi Decoction combined with prednisone and azathioprine will be in the case group and those taking prednisone and azathioprine will be in the control group. The whole study will last 48 weeks, including a 24-week observation period and a 24-week follow-up period. The primary outcome was complete response to therapy, defined as complete biochemical remission at the patient’s last visit of observation period and the absence of predefined steroid-specific side effects throughout treatment.

**Discussion:**

This trial will evaluate the efficacy and safety of Shu-Gan-Jian-Pi Decoction combined with prednisone and azathioprine on AIH patients. The achievement of this trial will provide evidence-based data for Shu-Gan-Jian-Pi Decoction, which could provide good benefits for AIH patients.

**Trial registration:**

Chinese Clinical Trial Registry: ChiCTR-OOC-15006155.

Registration date: 28 March 2015

## Background

Autoimmune hepatitis (AIH) is characterized by acute or chronic liver inflammation, which is considered to be associated with autoimmune response in liver cells. Previous studies [1] have proved that AIH can easily progress to cirrhosis or liver failure, and even some patients need for liver transplantation finally. It is reported that 40% of untreated severe AIH patients might die within 6 months, and those surviving commonly develop cirrhosis and subsequent complications of portal hypertension [2]. Moreover, it accounts for 2.6% of the transplantations in Europe [3] and 5.9% in the United States [4]. Due to this poor prognosis and the increasing detection rate, more and more studies focus on the management of AIH recently. It is well known that low dose prednisone plus azathioprine is the standard therapy for AIH, with alleviation of symptoms achieved in 60 to 80% of cases [5]. However, it must be noted that 50 to 86% of patients witness a recurrence of the disease after stopping medications, while 13% of patients stop the treatment too early due to the severity of the side effects and 9% of patients’ situation will deteriorate, even though they are administered medications according to guidelines [6]. Due to the high recurrence rate and the common side effects of corticosteroids, more and more AIH patients seek for traditional Chinese medicine (TCM) to manage their symptoms and reduce the side effects of corticosteroids in China.

In recent years, more and more researchers try to explore the efficacy and safety of combination treatment of steroids and Chinese herbal medicine in AIH patients. Fortunately, a number of clinical studies have proved the positive efficacy of TCM in Chinese AIH patients [7–9], such as alleviating liver inflammation; reducing side effects of corticosteroids and improving TCM signs and symptoms. But most of these studies were case reports or expert experience, or did not report strict inclusion and exclusion criteria, or had small sample size. Moreover, even the formulation of TCM was in the form of capsule or injection in some of these trials. Thus, current studies do not provide sufficient evidence to support the effectiveness of TCM in the treatment of AIH. So we have made a further research on the issue to explore whether the combination treatment of TCM and steroid standard therapy could improve the clinical management of AIH. Shu-Gan-Jian-Pi (SGJP) Decoction, made by famous traditional Chinese medicine doctor Chi Xiao-ling, is a popular used Chinese herbal formula in Guangdong province of China, which has demonstrated the effect of improving efficacy and reducing side effects of corticosteroids in AIH patients. This recipe, coming from the Golden mirror of medicine (a famous textbook of TCM), is widely used in treating chronic liver disease patients with pattern of live stagnation and spleen deficiency for more than 30 years. The results of previous clinical studies and animal experiments [10, 11] of SGJP Decoction provide the evidence that it has the effects of immunomodulatory and anti-liver-inflammation. However, previous clinical studies were retrospective study or focus on the patients with autoimmune diseases combined with hepatitis B, not on the AIH itself. Therefore, we conduct a prospective study to evaluate the efficacy and safety of SGJP Decoction combined with steroid standard therapy in AIH patients.

## Methods/design

### Study design

**Fig. 1:**
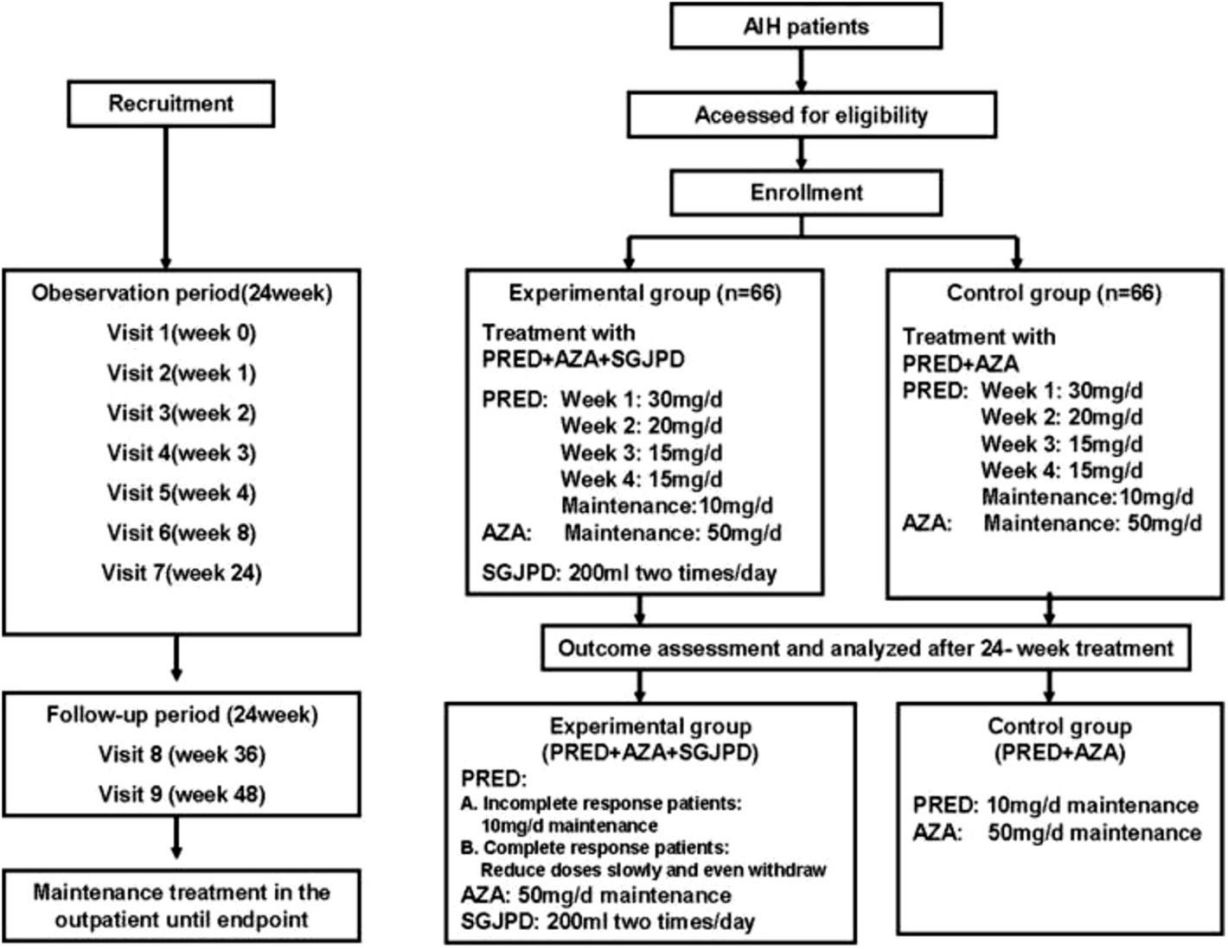
Flow chart of the current trial. PRED: Prednisone; AZA: azathioprine; SGJPD: Shu-Gan-Jian-Pi Decoction

This is a prospective, non-randomized, controlled study. One-hundred and thirty two AIH patients visited in Guangdong provincial hospital of Chinese medicine at the first time will be enrolled and divided into two groups with a case: control ratio of approximately 1:1. While patients in the control group received the steroid standard therapy, patients in the case group are treated with combination treatment of SGJP decoction and the steroid therapy. Both of the treatment and follow-up period will be for 24 weeks. The details of the flow chart of this trial are shown in Fig. 1.

### Patient population

A total of 132 AIH patients, who fulfill the screening criteria, is being enrolled and followed up between October 2015 and June 2017. According to the patients’ preference, the eligible AIH patients will be divided into two groups: the case group (to receive SGJP Decoction combined with low dose prednisone and azathioprine, *n* = 66) or a control group (to receive low dose prednisone and azathioprine, *n* = 66).

### Diagnostic criteria

The patients’ western medicine diagnostic criteria are based on AIH guideline which was established by American Association for the Study of Liver Diseases (AASLD) [12]. The TCM diagnosis of AIH patients is based on “Clinic terminology of traditional Chinese medicine medical diagnosis and treatment-Syndromes [13]”.

### Inclusion criteria

(1) All the patients had either a first diagnosis of AIH or were experiencing relapse after a previous diagnosis of AIH based on histology findings within 12 months before screening. (2) TCM syndrome type: liver stagnation and spleen deficiency. (3) Patients fulfill indications for treatment: serum aspartate aminotransferase (AST) ≥ 10-fold upper limit of normal (ULN) levels; or Serum AST ≥ 5-fold ULN and gammaglobulins ≥ twice ULN; or Bridging necrosis or multiacinar necrosis on histopathological examination (4) aged from 18 to 65 years.

### Exclusion criteria

(1) co-infection with hepatitis virus or human immunodeficiency virus; (2) evidence of advanced liver diseases, such as decompensated cirrhosis, severe hepatitis, or hepatic carcinoma; (3) evidence of serious uncontrollable diseases, such as heart, brain, kidney or gastrointestinal primary disease, etc.; (4) recent treatment with drugs with known liver toxicity; (5) pregnancy or lactation; (6) parenteral administration of blood or blood products within 6 months before screening.

### Interventions

While patients in the control group received the steroid standard therapy, patients in the case group are treated with combination treatment of SGJP decoction and the steroid therapy. The primary and secondary outcomes are assessed and analyzed after 24-week treatment.

### Steroid standard therapy

The steroid standard therapy, recommended by the AASLD AIH Guidelines [12], was as follows: prednisone (starting dose 30 mg/day, tapered to 10 mg/day) plus Azathioprine (administered at a dose of 50 mg/day). More details are shown in Fig. 1. According to the changes of patients’ conditions, doctors could adjust the specific agents and the doses, but the groups are not allowed to be changed until week 24.

### SGJP decoction

The formula comprises 11 herbs, including Radix Bupleuri (Chai Hu) 5 g, radices paeoniae alba (Bai Shao) 10 g, Tuckahoe (Fu Ling) 15 g, Turmeric Root Tuber (Yu Jin) 10 g, etc. The indication of the formula is liver stagnation and spleen deficiency pattern, including symptoms of fatigue, chest tightness, poor appetite, and abdominal distension, depressed, irritable and wiry and slippery pulse.

The procedure of preparing the SGJP decoction is as follows: firstly, all herbs are tipped into an earthenware pot with 1000 mL cold water and soaked for about half an hour. Secondly, give a boil with strong fire and then turn soft fire to simmer about 30 min until decoction reduces to 200 mL. Thirdly, the decoction is poured out and the same herbs with same water are boiled again for another 200 mL decoction over heat. Finally, these two times of decoction is mixed well together. Patients in the case group should take 200 mL of the decoction two times a day.

### Outcome measurements

#### Primary outcome

The primary outcome was complete response to therapy, defined as complete biochemical remission at the patient’s last visit of observation period and the absence of predefined steroid-specific side effects throughout observation period (moon face, central obesity, buffalo hump, diabetes and increased intraocular pressure, etc.). Biochemical remission was defined as complete normalization of aminotransferase levels including IgG.

#### Secondary outcomes

Secondary outcomes include the proportion of patients whose g-glutamyl transpeptidase (GGT) and total bilirubin (TBIL) levels decrease more than 50% after 4-week treatment as well as the GGT and TBIL normalization rate, the remission rate of patients’ clinical symptoms and the occurrence rate of the immunosuppressive agents’ side effects after 24-week treatment.

#### Safety outcomes

The results of a routine blood test, routine urine test, routine stool test, renal function test and electrocardiogram are assessed before and after the treatment to ensure the safety of this study. Further more, a urine pregnancy test or serum human chorionic gonadotropin test must be done before enrolling a women patient of childbearing age.

#### Data monitoring board

Two supervisors who are not members of the end-point committee should be assigned to contact and visit the researchers regularly to supervise the trial progress. Through this monitoring process, it can be ensured that it is conducted, recorded and reported in accordance with the protocol and standard operating procedures. In addition, the researchers should agree to cooperate with the supervisors to ensure that any problems found and resolved.

#### Ethics, consent and permission

Written informed consents will be obtained from all patients before enrollment. The study is being conducted in accordance with the Declaration of Helsinki (2008) [14] and has been approved by the ethics committees of Guangdong Provincial hospital of Chinese medicine. In addition, the study is registered with the Chinese Clinical Trial Registry (ChiCTR-OOC-15006155).

#### Sample size and statistical methods

This prospective study is intended to evaluate the efficacy and safety of the combination treatment of SGJP Decoction and steroid standard therapy on AIH. Since there is still no enough reliable data to accurately calculate the sample size for our study, 20 hepatology experts in Chinese medicine or integrative medicine with more than 20 years clinical experience were asked for advice. After two rounds of meetings with these experts, we agreed that 110 patients should be sufficient to gather enough information in order to plan such a clinical trial. And 132 cases are needed if there is a dropout rate of 15%.

The statistical analysis for this study will be performed using SPSS software version 19.0 (SPSS Inc., Chicago, IL, USA). Data will be presented as mean and standard deviation, or frequency and percentages. Comparisons will be conducted between the case group and the control group, also between pre-treatment and post-treatment in each group. For normally distributed variables, means will be compared by using t-tests (two-tailed), and nonparametric variables will be analyzed using Pearson chi-squared tests or Fisher exact tests. All *P*-values will be two-tailed. A P-value of less than 0.05 will be considered statistically significant.

## Discussion

AIH is known as a harmful disease with a high mortality which can easily progress to cirrhosis or liver failure. Prednisone plus azathioprine is considered the mainstay of therapy in the current recommendations for AIH therapy originate from this era. However, it does not provide good benefits for AIH patients because of its serious side effects. In this case, TCM has been popular used to treat AIH in China with good efficacy, low costs and small side effects. However, there is no rigorous and objective testing. What is more, Western medicine and TCM approaches have been used together to reach great effect worldwide [15]. Therefore, we conduct this study to explore whether the combination treatment of an empirical Chinese Herbal formula (SGJP Decoction) and steroids standard therapy could improve the clinical management of AIH.

This prospective study aims to explore the efficacy and safety of SGJP Decoction combined with steroid standard therapy on AIH. The achievement of this trial will provide evidence-based data for SGJP Decoction, which could provide good benefits for AIH patients. More over, the success of this study would represent justification and impetus for a large scale clinical study to further consolidate the evidence for the use of SGJP Decoction in AIH patients.

Our study also has several limitations that require consideration. Firstly, the case and control groups are not randomly assigned. However, we will use a new method of statistical analysis to make up for this shortage, the causal inference method, which has been used in previous randomized controlled trials [16]. In this method, propensity scores can balance the case and control group by limiting the confounding factors so that can help researchers to evaluate the interventions more accurately. Secondly, the sample size is still too small. However, the study produced critical data for sample size calculation of future study. And control of confounding variables, appropriate randomization, sufficient sample size and longer treatment period should be included in future study.

## Conclusions

This prospective study will verify that SGJP Decoction could reduce steroid dosing and side effects of steroid. On one hand, it will give evidence data for SGJP on AIH so that it could be applied widely and bring more good benefits for AIH patients. On the other hand, the findings will provide a basis and a new direction for further confirmatory studies.

### Trial status

This trial is currently recruiting participants.

## Abbreviations

AASLD: American Association for the Study of Liver Diseases
AIH: Autoimmune hepatitis
AST: Serum aspartate aminotransferase
AZA: Azathioprine
GGT: g-glutamyl transpeptidase
PRED: Prednisone
SGJP: Shu-Gan-Jian-Pi
SGJPD: Shu-Gan-Jian-Pi Decoction
TBIL: Total bilirubin
TCM: Traditional Chinese medicine
ULN: Upper limit of normal
